# Imbalanced biomedical data classification using self-adaptive multilayer ELM combined with dynamic GAN

**DOI:** 10.1186/s12938-018-0604-3

**Published:** 2018-12-04

**Authors:** Liyuan Zhang, Huamin Yang, Zhengang Jiang

**Affiliations:** School of Computer Science and Technology, Medical Imaging Engineering Laboratory, Changchun University of Science and Technology, No.7089, Weixing Road, Changchun, China

**Keywords:** Imbalanced data classification, Limited biomedical samples, High-dimensional feature, Multilayer ELM, Dynamic GAN

## Abstract

**Background:**

Imbalanced data classification is an inevitable problem in medical intelligent diagnosis. Most of real-world biomedical datasets are usually along with limited samples and high-dimensional feature. This seriously affects the classification performance of the model and causes erroneous guidance for the diagnosis of diseases. Exploring an effective classification method for imbalanced and limited biomedical dataset is a challenging task.

**Methods:**

In this paper, we propose a novel multilayer extreme learning machine (ELM) classification model combined with dynamic generative adversarial net (GAN) to tackle limited and imbalanced biomedical data. Firstly, principal component analysis is utilized to remove irrelevant and redundant features. Meanwhile, more meaningful pathological features are extracted. After that, dynamic GAN is designed to generate the realistic-looking minority class samples, thereby balancing the class distribution and avoiding overfitting effectively. Finally, a self-adaptive multilayer ELM is proposed to classify the balanced dataset. The analytic expression for the numbers of hidden layer and node is determined by quantitatively establishing the relationship between the change of imbalance ratio and the hyper-parameters of the model. Reducing interactive parameters adjustment makes the classification model more robust.

**Results:**

To evaluate the classification performance of the proposed method, numerical experiments are conducted on four real-world biomedical datasets. The proposed method can generate authentic minority class samples and self-adaptively select the optimal parameters of learning model. By comparing with W-ELM, SMOTE-ELM, and H-ELM methods, the quantitative experimental results demonstrate that our method can achieve better classification performance and higher computational efficiency in terms of ROC, AUC, G-mean, and F-measure metrics.

**Conclusions:**

Our study provides an effective solution for imbalanced biomedical data classification under the condition of limited samples and high-dimensional feature. The proposed method could offer a theoretical basis for computer-aided diagnosis. It has the potential to be applied in biomedical clinical practice.

## Background

In the biomedical domain, machine learning techniques can make computer-aided diagnosis (CAD) [1] more intelligent in diagnoses of breast cancer, liver disorder, and other diseases. While imbalanced class distribution frequently occurs in real-world biomedical datasets, which causes the loss of essential pathological information from abnormal class [2]. Indeed, the misdiagnosis of abnormal class is more severe than that of a normal class in medical disease diagnosis [3]. Additionally, the training set sometimes contains high-dimensional feature and small samples. These factors further result in a lower classification accuracy of abnormal class and incorrect diagnosis result [4]. Therefore, establishing an effective classification model is an urgently necessary task for limited and imbalanced biomedical dataset.

To solve class-imbalanced classification problem, many studies [5–12] have been proposed. These methods mainly focus on three strategies: the algorithm level, the data level, and hybrid method. For the first strategy, the algorithm-based method often needs to amend the model parameters. Among numerous classifiers, ELM is famous owing to its analytical solution and fast learning speed, which is applicable to the engineering applications [13]. Various scholars have proposed some improved ELM models for imbalanced data classification [14–16]. So far, the weighted extreme learning machine (W-ELM) [17] is the most representative learning method for the class-imbalanced classification. The samples belonging to different classes are assigned different weights. This method attaches great importance to the minority class samples and alleviates the bias towards the majority class. A computationally efficient cost-sensitive method [18] has been developed by integrating a cost factor into the fuzzy rule-based classifier. The misclassified cost of majority class is set to one, while the penalty value of minority class equals to the imbalanced ratio. It is well suitable for a larger dataset. To extract hidden pathological features, forming a deep representation may be more meaningful [19]. Hierarchical ELM (H-ELM) [20] as a multilayer neural network has stable hierarchical structure. And it can produce a better feature representation by unsupervised feature learning. In view of the second strategy, the data-based method [21–24] concentrates on generating new samples for minority class (oversampling) or removing samples from majority class (undersampling). Resampling techniques are often employed as a preprocessing process. Different from cost-sensitive method, it is much easier to be implemented. The synthetic minority oversampling technique (SMOTE) [25] is a typical method. It creates synthetic samples to oversample the minority samples rather than mere data duplicating, thus avoiding the overfitting. Also, it is more helpful in recognizing outliers. Despite the goodness, this resampling method is prone to neglect the sample distribution and lead to the information loss.

The last strategy is the widely-employed hybrid method. Apart from the preprocessing methods, a better classification algorithm is beneficial for class-imbalanced classification task. For example, Yu et al. [26] proposed a combination method of asymmetric bagging ensemble classifier and feature subspace (Bagging-FSS). This method adopts random projection to establish the relationship between feature selection and ensemble classifier. The single classifier performance is improved by combining advantages of data pre-processing and ensemble learning methods in practical tasks. Similarly, Krawczyk et al. [27] combined the boosting scheme and evolutionary undersampling (EUS) technology for imbalanced classification of breast cancer malignancy. The usage of EUS allows selecting the most representative samples for boosting classifier, thereby improving the diversity of base classifiers. In fact, if the training sample is limited, this model will be difficult to guarantee the diversity of base classifiers. Moreover, this ensemble learning method largely depends on the performance of base classifier. In [28], synthetic minority oversampling technique and ELM (SMOTE-ELM) are integrated to provide an efficient solution for the imbalanced data classification. To produce a balanced dataset, the distribution of majority class samples is taken into consideration. Then, the oversampling of minority samples is conducted. SMOTE-ELM method has the lower bound of model reliability and reduces the information loss of majority samples. However, when addressing smaller dataset, particularly less training samples, the aforementioned works face some issues. How to establish the quantitative relationship between feature extraction and model selection should be considered to reduce manual parameters tuning. For this purpose, a specifically designed method to address the imbalanced biomedical data classification has important meanings in medical intelligent diagnosis.

In this paper, a self-adaptive multilayer ELM model with dynamic generative adversarial net (GAN) (for short PGM-ELM) is proposed to solve the class-imbalanced classification problem. The proposed method makes biomedical data classification more efficient and robust in the context of small-data and high-dimensional feature. The main contributions of this paper are summarized as follows: Principal component analysis (PCA) is used to remove irrelevant and redundant features from raw feature set, thereby extracting more effective features; Dynamic GAN is introduced to generate the realistic-looking minority class samples and balance the class distribution, thus alleviating effect of the imbalanced dataset and avoiding overfitting; The analytic expression for numbers of hidden layer and node is determined by establishing the quantitative relationship among the changes of imbalance ratio, the sample distribution, and the hyper-parameters of model. This provides a solution for reducing the parameter sensitivity of multilayer ELM. The effectiveness of the PGM-ELM model is validated and evaluated on four biomedical datasets. The obtained experimental results can help guide us to construct the optimal classification model for practical biomedical applications.

The remaining of the paper is organized as follows. “Related works” section simply introduces the basic principles of hierarchical ELM and classical GAN. Then, the detailed process of the proposed method is described in “Methods” section. Afterwards, the dataset description, evaluation metrics, and experimental results are presented in “Results” section. Comparative analyses of the proposed method with other state-of-the-art methods are given in “Discussions” section. Finally, “Conclusions” section provides the conclusion and future research directions of this paper.

## Related works

Some variants of the ELM model have been employed effectively. Here the basic principles of H-ELM and classic GAN are briefly described. They can contribute to the solving of imbalanced biomedical data classification.

### Hierarchical extreme learning machine framework

In network structure of H-ELM, the original input is decomposed into multiple hidden layers. The output of the previous layer is regarded as the input of the current one. The learning of hidden layer represents more abstract information. By doing so, the hidden information can be exploited for deeper feature representation.

**Fig. 1:**
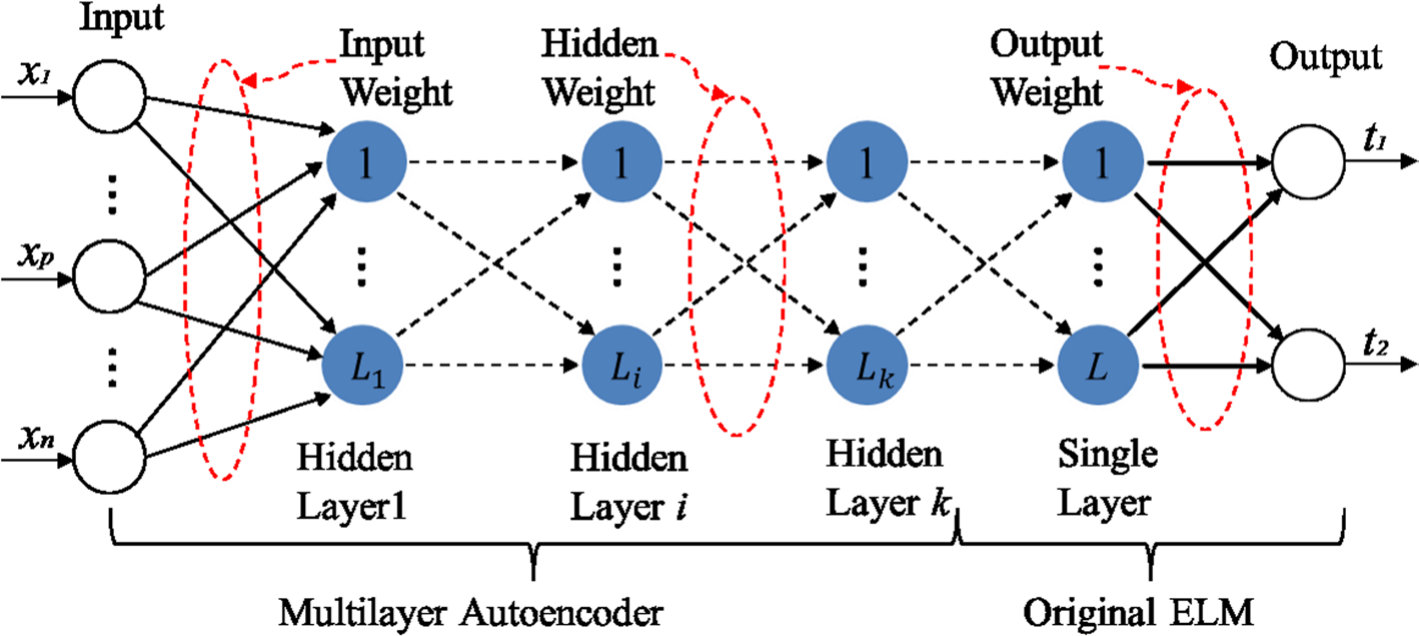
The network structure of H-ELM: *k*-layer feature learning with *L* hidden-node ELM feature classification

Assume that we have a training set ${\left\{ ({x_i,t_i})\right\} }_{i=1}^N$, where $x_i$ denotes the input node *i*, and $t_i$ stands for the output of the *i*th sample. A single hidden layer feedforward neural network with *L* hidden nodes is used to fit *N* training samples. Then, the corresponding output function of ELM can be expressed as [29]

1 $$\begin{aligned} f\left( x \right) = h\left( x \right) \beta = h\left( x \right) \left( {\frac{\mathbf{I }}{C} + \mathbf{HH }^T } \right) ^{ - 1} \mathbf{H }^T \mathbf{T }, \end{aligned}$$

where $\mathbf{H} = \left[ {h\left( {x_1 } \right) , \ldots ,h\left( {x_N } \right) } \right] ^T$ is the randomized output matrix of hidden layer, and $\mathbf{T} = \left[ {t_1 , \ldots ,t_N } \right] ^T$ is the target matrix of the output layer. $\beta$ denotes the connection weight from a hidden layer node to each output node. *C* is a regularization coefficient. $\mathbf {I}$ is a unit matrix. The input weight and bias will be assigned randomly. Desired outputs of minority and majority classes are set to 1 and 0. Figure 1 shows the basic network structure of H-ELM, which consists of two separate parts: unsupervised and supervised training.

The first phase is unsupervised feature learning by ELM-based autoencoder (ELM-AE) [30]. ELM-AE based $\ell _1$-norm optimization is employed to form a multi-layer feature learning model. By recovering the input data as much as possible, new features can be learned to represent the input data. A fast iterative shrinkage-thresholding algorithm (FISTA) [31] is utilized to obtain weight $\beta$ of each hidden layer. The optimization model of ELM-AE is given by

2 $$\begin{aligned} O_\beta = \arg \min \left\{ {\left\| {\mathbf{H }\beta - \mathbf{X }} \right\| ^2 + \left\| \beta \right\| _{\ell _1 } } \right\} , \end{aligned}$$

where $\mathbf{X}$ is the original input data. $\mathbf{H}$ represents the random initialized output.

Next, the second phase is supervised feature classification. The original ELM is performed for final decision making. The output of the H-ELM is calculated by using the last layer output of the ELM-AE as the input of the parallel ELM. Mathematically, the output of each hidden layer can be represented as

3 $$\begin{aligned} \mathbf{H }_i = g\left( {\mathbf{H }_{i - 1} \cdot \beta } \right) , \end{aligned}$$

where $\mathbf{H }_i \left( i \in \left( {1, \ldots ,K} \right) \right)$ is the output of the $i\hbox {th}$ hidden layer. $g(\cdot )$ denotes the activation function of the hidden layers, and $\beta$ represents the output weight. Here, the node number $L_{k}$ of the $k\hbox {th}$ hidden layer equals to the node number $L_{k-1}$ of the $(k-1)\hbox {th}$ hidden layer. Different from deep back propagation (BP) network, all hidden neurons in H-ELM as a whole are not required to be iteratively tuned. The parameter of the last hidden layer will be adjusted no longer.

### Generative adversarial net

**Fig. 2:**
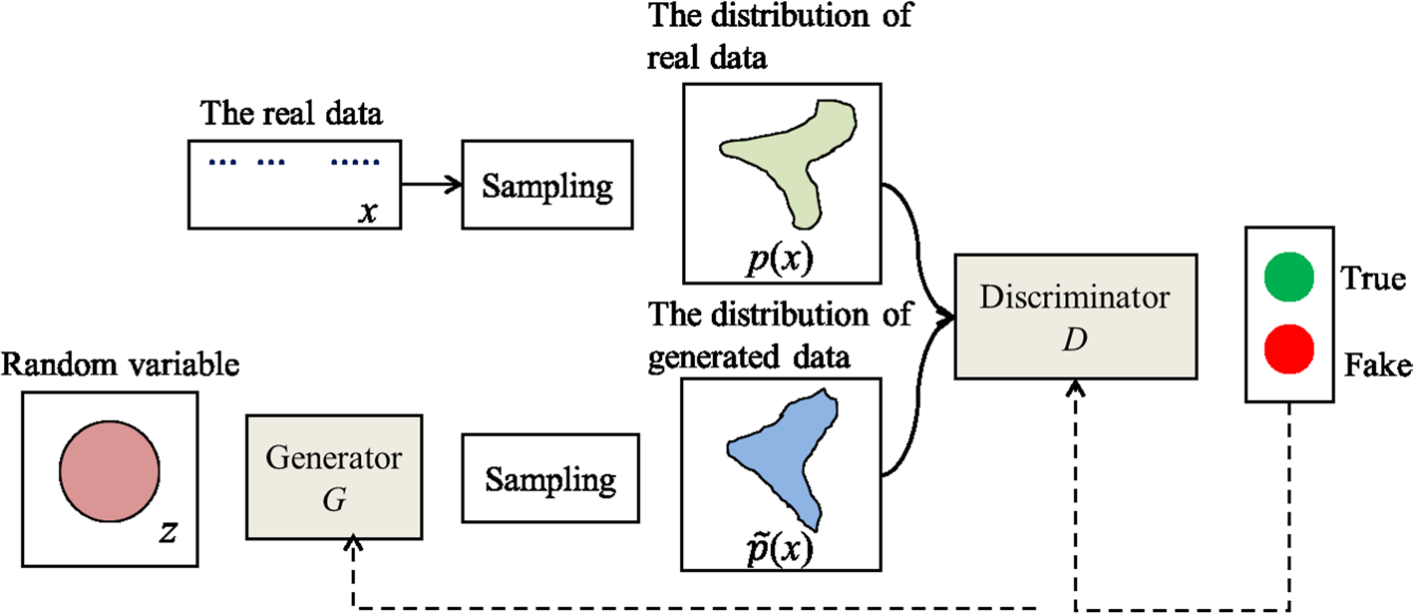
The data generation framework of GAN

GAN [32] is a combination method of simulation and unsupervised learning, and it largely depends on the adversarial relationship among competitive neural networks. GAN can generate entirely new data like the observed data based on the probability distribution model. Figure 2 presents the whole data generation process. GAN simultaneously trains the generative model *G* and the discriminative model *D* by playing a non-cooperative game. *G* can capture the data distribution to generate samples, while *D* assists *G* to classify these samples as true or fake. By discriminator *D* to optimize, the parameters of *G* are adjusted to make the probability distribution $\tilde{p}(x)$ and the real data distribution *p*(*x*) as close as possible.

This process can be expressed by minimizing an objective function. The overall objective function of GAN model is given as follows

4 $$\begin{aligned} \min _{G}\max _{D}V(D,G)=E_{x\sim p_{data}(x)}\bigl [\log {D(x)}\bigr ]+E_{z\sim p_{z}(z)}\bigl [\log {(1-D(G(z)))}\bigr ], \end{aligned}$$

where $p_{data}(x)$ is the distribution of the training set. $p_{z}(z)$ is the distribution of noise. *E* denotes the expectation. If the generator *G* is fixed, the optimal discriminator *D* is depicted by the following formula.

5 $$\begin{aligned} D_{G}^*(x)=\frac{p_{data}(x)}{p_{data}(x)+p_{g}(x)}, \end{aligned}$$

where $p_{g}(x)$ expresses the probability distribution of the generator. The training objective for *D* can be interpreted as maximizing the log-likelihood for estimating the conditional probability $P(Y=y|x)$. The *Y* makes clear whether the *x* comes from the real data or the generated data. Therefore, the minimax game in Eq. (4) can be rewritten as

6 $$\begin{aligned} \max _{D}V(G,D)=E_{x\sim p_{data}} \left [\log {\frac{p_{data}(x)}{p_{data}(x)+p_{g}(x)}} \right]+E_{x\sim p_g} \left [\log {\frac{p_{g}(x)}{p_{data}(x)+p_{g}(x)}}\right]. \end{aligned}$$

*G* and *D* will reach a balance after conducting several times training, that is $p_g=p_{data}$. The discriminator is incapable to distinguish the difference between two distributions, such that $D_{G}^*(x)=1/2$ [33].

## Methods

**Fig. 3:**
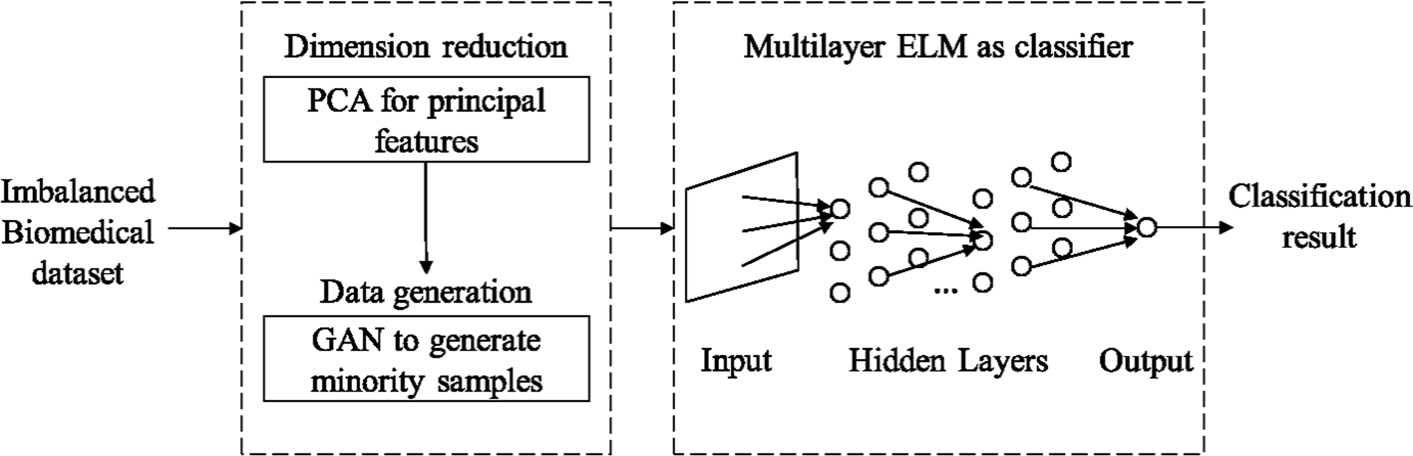
The overall framework of the PGM-ELM method

Throughout this paper, aiming at the limited and imbalanced biomedical data, a hybrid PGM-ELM classification model is proposed. Figure 3 shows the whole process of the proposed method. In Fig. 3, the model first employs PCA to extract the principal features and reduce dimensionality. Afterwards, we use GAN to dynamically generate real minority class samples, thus balancing the class distribution. Lastly, considering the numbers of samples and features, once the quantitative relationship between the imbalance ratio and the hyper-parameters of multilayer ELM is established. A self-adaptive PGM-ELM classification model is constructed for imbalanced classification.

For a given training set with *N* samples $DS={\left\{ ({\mathbf{x}_i,y_i})\right\} }_{i=1}^N$, $\mathbf{x}_i$ denotes the feature vector of the $i\hbox {th}$ sample, and $y_i$ is the class label of the $i\hbox {th}$ sample. In our study, the medical diagnosis with or without lesions is identified as a binary classification problem. For convenience, $N^+$ represents the number of the minority class samples, and $N^-$ represents the number of the majority class samples. $N=N^{-}+N^+$ is the total number of all samples in training set.

### Principal features extraction

Most of original biomedical datasets have lots of noise and redundant features. PCA is adopted to remove the irrelevant and redundant information [34]. For the original feature set $X=\left\{ x^{(1)},x^{(2)},\ldots ,x^{(M)}\right\}$, the matrix $\tilde{X}$ is obtained through standardized processing. This transform relation is given by

7 $$\begin{aligned} {\tilde{x}}^{(i)}=\frac{x^{(i)}-\mu ^{(i)}}{\delta ^{(i)}}, \end{aligned}$$

where ${\tilde{x}}^{(i)}$ is the *i*th feature of standardized matrix. $x^{(i)}$ is the *i*th sample in original feature set. $\mu ^{(i)}$ and $\delta ^{(i)}$ are the mean value and the variance of the original features. The covariance matrix is calculated as follows

8 $$\begin{aligned} R={{\tilde{X}}^T{\tilde{X}}}/{(M-1)}. \end{aligned}$$

The eigenvalue decomposition is applied to solve the eigenvalues and corresponding eigenvectors of the covariance matrix. The eigenvalues are arranged from large to small, and the contribution rate is computed. The formula is described as follows

9 $$\begin{aligned} \alpha = {{\sum\limits_{{k = 1}}^{r} {\lambda _{k} } } \mathord{\left/ {\vphantom {{\sum\limits_{{k = 1}}^{r} {\lambda _{k} } } {\sum\limits_{{k = 1}}^{{M - 1}} {\lambda _{k} } }}} \right. \kern-\nulldelimiterspace} {\sum\limits_{{k = 1}}^{{M - 1}} {\lambda _{k} } }}, \end{aligned}$$

where $\lambda _k$ denotes the $k\hbox {th}$ eigenvalue. The threshold of cumulative contribution rate of the eigenvalue is selected as 85%. When the proportion of the largest $M'$ eigenvalues is greater than this threshold, $M'$ is viewed as the number of the principal components. By calculating the product of the standard feature matrix and eigenvector, we get the corresponding principal component vector, which is expressed as follows

10 $$\begin{aligned} z^{\left( i \right) } = \sum \limits _{j = 1}^{M'} {{{\tilde{x}}}^{\left( j \right) } {\varvec{\eta }}_i^T }, \end{aligned}$$

where ${\varvec{\eta }}_i$ represents the standard orthogonal eigenvectors corresponding to the $i\hbox {th}$ eigenvalues. $Z=\left\{ z^{(1)},z^{(2)},\ldots, z^{(M')}\right\}$ is new feature set after analyzing the principal components.

### Samples generation

From the perspective of the data, dynamic GAN generates new samples to change the imbalanced ratio. To fully make use of the data distribution, all minority class samples as a whole chunk are input into GAN model. And then, dynamic GAN is executed multiple times to balance class samples. It is worthy note that the execution number of GAN is set to $num=\left\lfloor {\frac{{N^ - }}{{N^ + }}} \right\rfloor$ according to initial imbalanced ratio, where $\lfloor \cdot \rfloor$ is on behalf of the round down. That is to say, the samples generation procedure using GAN is repeated until the imbalanced ratio is closer to 1. By doing so, the class distribution is balanced as much as possible.

For the minority class samples $\mathbf {X^+}$, the initial condition is noise $\mathbf {Z}$ with the same size as the whole target fragment. The objective function of GAN can be depicted by the following formula.

11 $$\begin{aligned} \begin{aligned} \min _{G}\max _{D}V(D,G)=\,&\,E_{\mathbf{x_k^+}\sim p_{data}(num\cdot \mathbf{X^+})}\left [\log {D\left(\left\lfloor {\frac{N^-}{N^+}} \right\rfloor \cdot \mathbf{X^+ }\right)}\right ]\\&+E_\mathbf{z\sim p_\mathbf{z}(\mathbf z)}\left [\log {(1-D(G(\mathbf Z)))}\right ]. \end{aligned} \end{aligned}$$

The optimal discriminator *D* equals to $\frac{p_{data}(\mathbf X^+)}{p_{data}(\mathbf X^+)+p_g{(\tilde{\mathbf{X}}^+)}}$. $p_g{(\tilde{\mathbf{X}}^+)}$ denotes the distribution of generated data. The discriminator *D* can be updated by whole target segment.

12 $$\begin{aligned} \nabla _{\theta _d } \frac{1}{{num \cdot N}}\sum \limits _{i = 1}^{num \cdot N} {\left[ {\log D(x_i ) + \log (1 - D(G(z_i )))} \right] }, \end{aligned}$$

where, $x_i$ and $z_i$ denote the samples of $\mathbf X^+$ and $\mathbf Z$. $\theta _d$ is the parameter of discriminator *D*. Generator *G* is updated by

13 $$\begin{aligned} \nabla _{\theta _g } \frac{1}{{num \cdot N}}\sum \limits _{i = 1}^{num \cdot N} {\left[ {\log (1 - D(G(z_i )))} \right] }, \end{aligned}$$

where $\theta _g$ is the parameter of generator *G*. If *G* recovers data distribution, and *D* equals to 0.5 in any instance, the new samples $\tilde{\mathbf{X}}^+$ will be generated. The sample number of the training set is increased to $N'=\left\lfloor {\frac{N^ - }{N^ + }} \right\rfloor \cdot {N^ +} + N^-$. $IR=\frac{{N^ + }}{{N^ - }}$ is initial imbalanced ratio of the training set, while $IR'=\left\lfloor {\frac{N^ - }{N^ + }} \right\rfloor \cdot {N^ +}$ represents new imbalanced ratio after samples generation. For clear representation, the change of imbalanced ratio $\Delta IR$ can be obtained as follows

14 $$\begin{aligned} \Delta IR = IR' - IR = \frac{{\left\lfloor {\frac{{N^ - }}{{N^ + }}} \right\rfloor \cdot N^ + }}{{N^ - }} - \frac{{N^ + }}{{N^ - }}\mathrm{{ = }}\frac{{\left( {\left\lfloor {\frac{{N^ - }}{{N^ + }}} \right\rfloor \mathrm{{ - }}1} \right) \cdot N^ + }}{{N^ - }}. \end{aligned}$$

### Self-adaptive multilayer ELM modeling

In last phase of the PGM-ELM, using the multilayer ELM model is to classify the balanced dataset. The network structure of the classification model is first determined. In fact, multilayer ELM is sensitive to the numbers of hidden layer and node. Sometimes it is difficult for users to specify an appropriate number of nodes without prior knowledge. If the number of nodes is too small, the classifier is unable to learn feature well, causing the under-fitting performance. If the number of nodes is too big, the time complexity of the network structure will be increased. Generally, it is related to the numbers of sample and feature. Therefore, the change of the imbalanced ratio and the number of new features are considered in our multilayer ELM model. Mathematically, the number of hidden nodes is obtained by

15 $$\begin{aligned} P = \left\lceil {\left( {1 - \Delta IR} \right) \times \frac{N}{M} + \Delta IR \times \frac{{N'}}{{M'}}} \right\rceil . \end{aligned}$$

Simultaneously, the number of hidden layers is determined by

16 $$\begin{aligned} Q = \left\lceil {\Delta IR \times M'} \right\rceil , \end{aligned}$$

where $\left\lceil {\cdot } \right\rceil$ shows the round up.

It can be found that, on the one hand, the bigger the change of imbalanced ratio is, the greater the number of hidden layers is. On the other hand, the more numbers of the feature and generated samples are, the larger the number of hidden nodes is. This specific relationship can self-adaptively adjust the parameters of model for different datasets. After that, the designed network is learned layer by layer using the M–P generalized inverse. And the functional relationship of each layer is achieved as follows

17 $$\begin{aligned} {\varvec{\beta }} = \mathbf{H}_Q ^T \left( {\frac{\mathbf{I}}{C} + \mathbf{H }_Q \mathbf{H }_Q ^T } \right) ^{ - 1} \mathbf{T }_Q, \end{aligned}$$

where $$\mathbf{H }_Q = \left[ {\begin{array}{*{20}c} {g(a_1 \cdot x_1 + b_1 )} &{} \ldots &{} {g(a_L \cdot x_1 + b_P )} \\ \vdots &{} \ldots &{} \vdots \\ {g(a_1 \cdot x_{N'} + b_1 )} &{} \ldots &{} {g(a_L \cdot x_{N'} + b_P )} \\ \end{array}} \right] _{N' \times P}$$ is the output matrix of the *Q*th hidden layer. *a* is the orthogonal random weight vector between input nodes and hidden nodes. *b* is the orthogonal random threshold of the hidden neurons. The sigmoid function is selected as the activation function $g(\cdot )$. This function expression is

18 $$\begin{aligned} g\left( u \right) = \frac{1}{{\left( {1 + \exp \left( { - au} \right) } \right) }}. \end{aligned}$$

Finally, the output matrix $\varvec{\beta }$ is obtained, and the entire hybrid model is established. Pseudo-code description for the process of hybrid approach is shown as Algorithm 1.

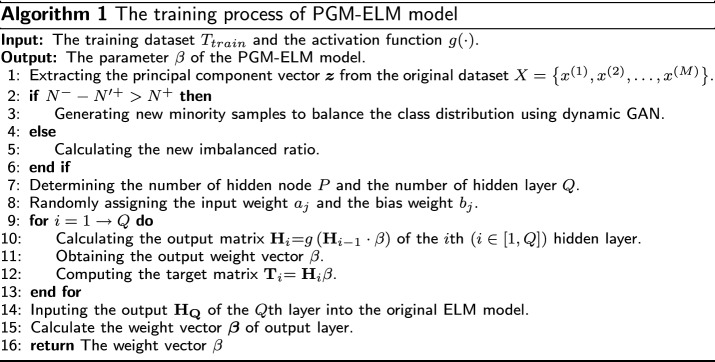



## Results

In this section, to validate the effectiveness of the proposed PGM-ELM method, extensive experiments have been performed. We first describe four real-world imbalanced biomedical datasets derived from the UCI machine learning repository [35]. Then we present the classification results of our method. Also, the obtained results are discussed adequately. Our experimental computer configurations are listed as follows: Intel(R) dual-core, 3.20 GHz, 8 GB RAM with Windows 7 Operating System. All algorithms in this study are programmed with MATLAB R2014a.

### Datasets description

For constructing a small training sample set, each dataset are divided into the training and test sets via a random sampling process. The breast cancer diagnostic dataset provides information on the discrimination of benign and malignant. Each instance has one ID number, 30 real value variables and one diagnosis label. The Indian liver dataset describes liver patient or not, which is made up of two patient information, eight real-valued features and a class label. The diabetic retinopathy Debrecen dataset with 19 numerical features contains the sign of diabetic retinopathy or not. The Pima diabetes dataset collects pathologic data from diabetes patients, including eight real-valued features and a class label. Table 1 summarizes the detailed information of the four biomedical datasets.

**Table 1:** Description of the experimental datasets

Datasets	Attributes	Minority	Majority	Imbalance ratio	Training samples	Test samples
Breast cancer	32	212	357	0.59	100	469
Liver patient	11	26	224	0.12	50	200
Diabetic retinopathy	20	100	300	0.30	100	300
Pima diabetes	9	117	517	0.23	200	434

From Table 1 we can see that these four datasets are imbalanced since the imbalance ratios are much less than 1. Besides, they have different feature dimensionalities and smaller instances. It is noticeable that all datasets should be normalized to facilitate processing. Furthermore, only real-valued features are used as the input of the model in all experiments. Considering the fact that the distinction between normal and abnormal is a typical two-class classification task, so the labels containing majority and minority classes are specified as 0 and 1, respectively.

### Performance evaluation metrics

In order to evaluate the classification performance of the proposed model, there are several commonly considered measurement criteria that can be used in imbalanced classification task [36]. First, Table 2 gives the confusion matrix of a two-class problem for explaining the performance measures. TP and TN are the numbers of correctly classified positive and negative samples, respectively. FP and FN are the numbers of the misclassified negative and positive samples, respectively. The confusion matrix gives the quantitative classification results on each dataset.

**Table 2:** Confusion matrix for a two-class problem

	Positive prediction	Negative prediction
Positive class	True positive (TP)	False negative (FN)
Negative class	False positive (FP)	True negative (TN)

And then, receiver operator characteristic (ROC) is a graphical method to intuitively show the compromise between the true positive rate and false positive rate for the classification models. Area under the ROC curve (AUC) can describe the performance of classifiers in different decision thresholds. The AUC value is larger, the better the performance of classifier is. G-mean is a popular measure to indicate the geometric mean of sensitivity and specificity. F-measure is the harmonic mean of precision and recall. They can be effective to evaluate generalization performance than overall classification accuracy, and their definitions are expressed as follows.

19 $$\begin{aligned} G\text{-}mean= \sqrt{TPR \cdot TNR}, \end{aligned}$$

20 $$\begin{aligned} F\text{-}measure= & {} \frac{{2 \times \mathrm{Precision} \times \mathrm{Recall}}}{{\mathrm{Precision + Recall}}}, \end{aligned}$$

where, true positive rate (TPR) represents the proportion of positive samples to be correctly classified as positive class, whose definition is the same as Recall. True negative rate (TNR) indicates the proportion of negative samples to be correctly classified as negative class. Precision denotes the proportion of positive samples to be correctly classified and all positive samples. They are defined in the following.

21 $$\begin{aligned} \mathrm{TNR}=\, & {} {\frac{{\mathrm{TN}}}{{\mathrm{FP } + \mathrm{TN }}}}. \end{aligned}$$

22 $$\begin{aligned} \mathrm{TPR}=\, & {} \mathrm{Recall} = {\frac{{\mathrm{TP}}}{{\mathrm{TP } + \mathrm{FN}}}}. \end{aligned}$$

23 $$\begin{aligned} \mathrm{Precision }=\, & {} \frac{{\mathrm{TP}}}{{\mathrm{TP } + \mathrm{FP }}}. \end{aligned}$$

### The result analysis of dynamic GAN

**Fig. 4:**
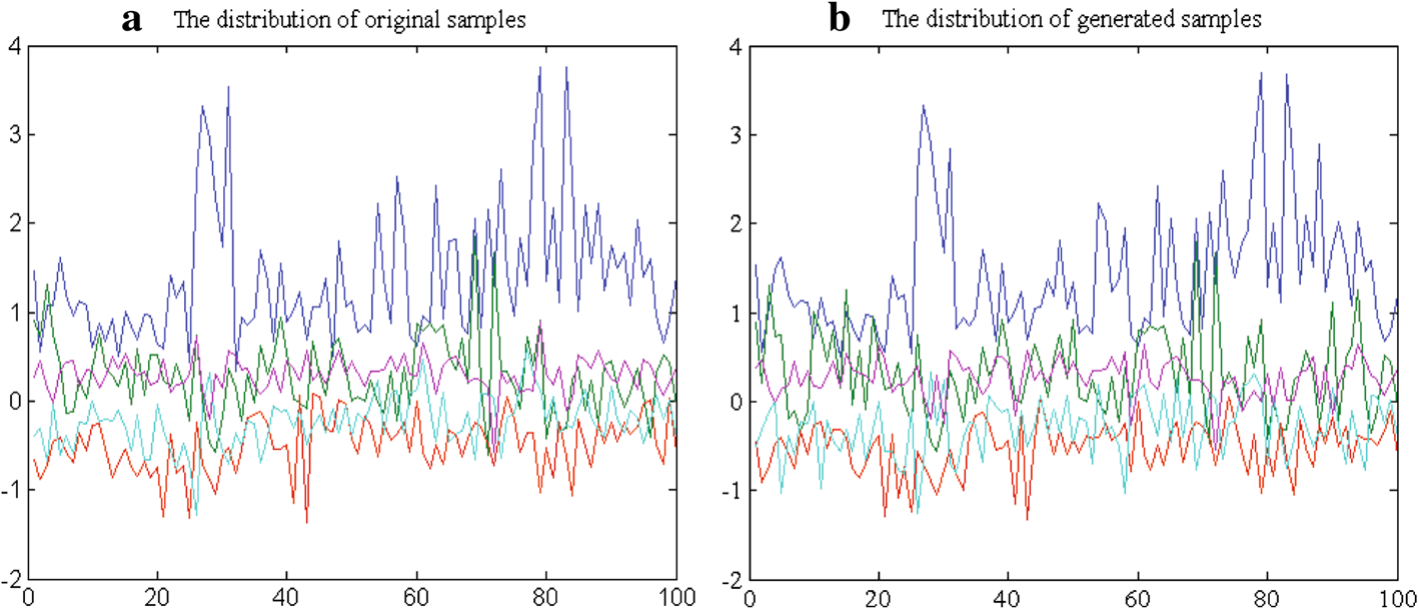
The comparison result of samples distribution on breast cancer dataset. **a** The distribution of original samples. **b** The generated samples by dynamic GAN

First of all, the principal components of original feature set are extracted from a given imbalanced training set by using PCA. Thereafter, new balanced dataset are achieved after generating minority class samples using dynamic GAN. In the network structure of dynamic GAN, several appropriate parameters are selected to generate realistic minority class samples. The number of hidden nodes is set to 100. The learning rate is set to 0.01. Dropout fraction of discriminator *D* and generator *G* are set to 0.9 and 0.1, respectively. The activation function of GAN is given as follows: the generator *G* uses ReLU and Sigmoid, while the discriminator *D* employs Maxout and Sigmoid. Figure 4 depicts the comparative distributions of the original samples and the generated samples after performing the dynamic GAN.

In Fig. 4, five different colors represent five principal components after performing PCA. There are 100 minority class samples derived from breast cancer dataset. In general, similar dataset should be represented by similar distribution. We can easily observe that, the distribution of the generated samples is consistent with the original sample distribution. This visually proves that the dynamic GAN is capable to capture the distribution of actual data to generate convincing samples, thus balancing the class distribution and avoiding the overfitting.

To quantify the quality of generated data, we compute the dissimilarity between the distributions of generated data and original data by means of kernel maximum mean discrepancy (MMD). Kernel MMD [37] is a popular sample-based evaluation metric for quantitatively evaluating GANs model. A lower MMD means that the distribution of generated data is consistent with that of the real data. Table 3 reports the comparison results of Kernel MMD on four datasets. All MMD values are calculated over 50, 100 and 200 samples generated by dynamic GAN. In Table 3, as increasing the number of samples, the smaller the MMD value is, the higher the quality of generated samples is. Based on this quantitative result, we can conclude that the dynamic GAN can capture the training data distribution. GAN can be appropriate for producing samples without the information loss of majority class in class-imbalanced classification.

**Table 3:** Comparison result of Kernel MMD on four test sets

The dataset	50 samples	100 samples	200 samples
Breast cancer	0.104	0.096	0.089
Liver patient	0.181	0.118	0.124
Diabetic retinopathy	0.219	0.143	0.078
Pima diabetes	0.072	0.057	0.060

### Analysis of the classification results

In order to examine the classification results of PGM-ELM against other constructive algorithms: W-ELM, SMOTE-ELM, and H-ELM. We give the corresponding results of these algorithms on four biomedical datasets. Considering fact that the weight of ELMs model is randomly chosen, four methods are ran 20 independent monte carlo trials. The final result is from the average of the 20 results. For fair comparison, these methods use same sigmoid activation function for learning.

**Fig. 5:**
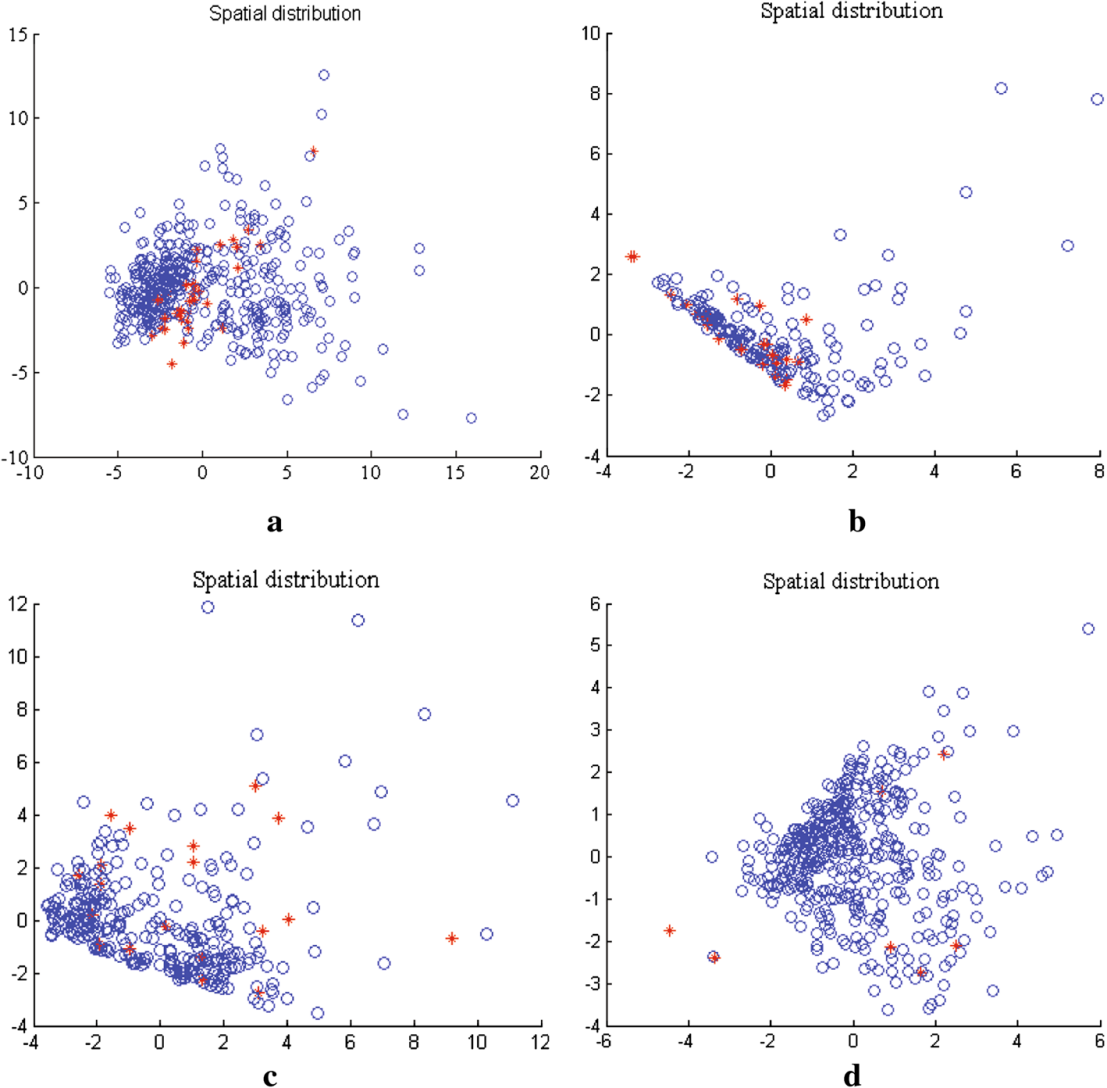
Spatial distribution of sample using PGM-ELM. (Blue circles describe the correctly classified samples, while the red stars mean the misclassified samples.) **a** Breast cancer dataset. **b** Liver patient dataset. **c** Diabetic retinopathy dataset. **d** Pima diabetes dataset

Consequently, Fig. 5 displays the spatial distribution of classification results on four datasets after performing one monte carlo trial. The correctly classified samples and the misclassified samples are visualized. From Fig.  5 can be seen that the correctly classified samples are much more compared to the misclassified ones on each dataset. Obviously, Pima diabetes dataset yields the best classification result of PGM-ELM model. And its misclassified samples number is much less than those of other datasets. This reflects better classification ability of the PGM-ELM for most of biomedical datasets.

**Fig. 6:**
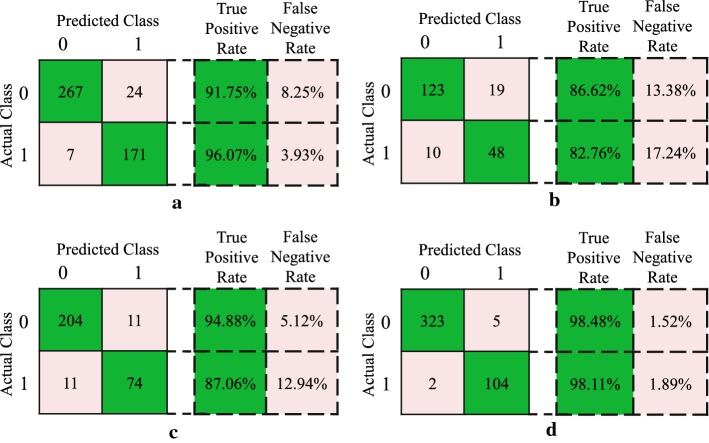
Confusion matrix of PGM-ELM on four biomedical datasets. **a** Breast cancer dataset. **b** Liver patient dataset. **c** Diabetic retinopathy dataset. **d** Pima diabetes dataset

Apart from the spatial distribution results, the result of confusion matrix (two-class case: 0 for majority class and 1 for minority class) on four biomedical datasets is presented in Fig. 6. The numbers of correctly classified and misclassified samples are shown. Corresponding true positive rate (TPR) and false negative rate (FNR) are computed. Taking breast cancer dataset as an example, given a classification of the minority class 1, 171/178 will be correct (class 1). Moreover, the number of misclassified minority sample is smaller than the misclassified rate of the majority class. It can be seen that most of predicted samples are classified as actual class on each dataset. Therefore, the proposed PGM-ELM significantly improves the classified rate of minority class samples. This reflects a superior classification capacity for imbalanced biomedical dataset.

**Fig. 7:**
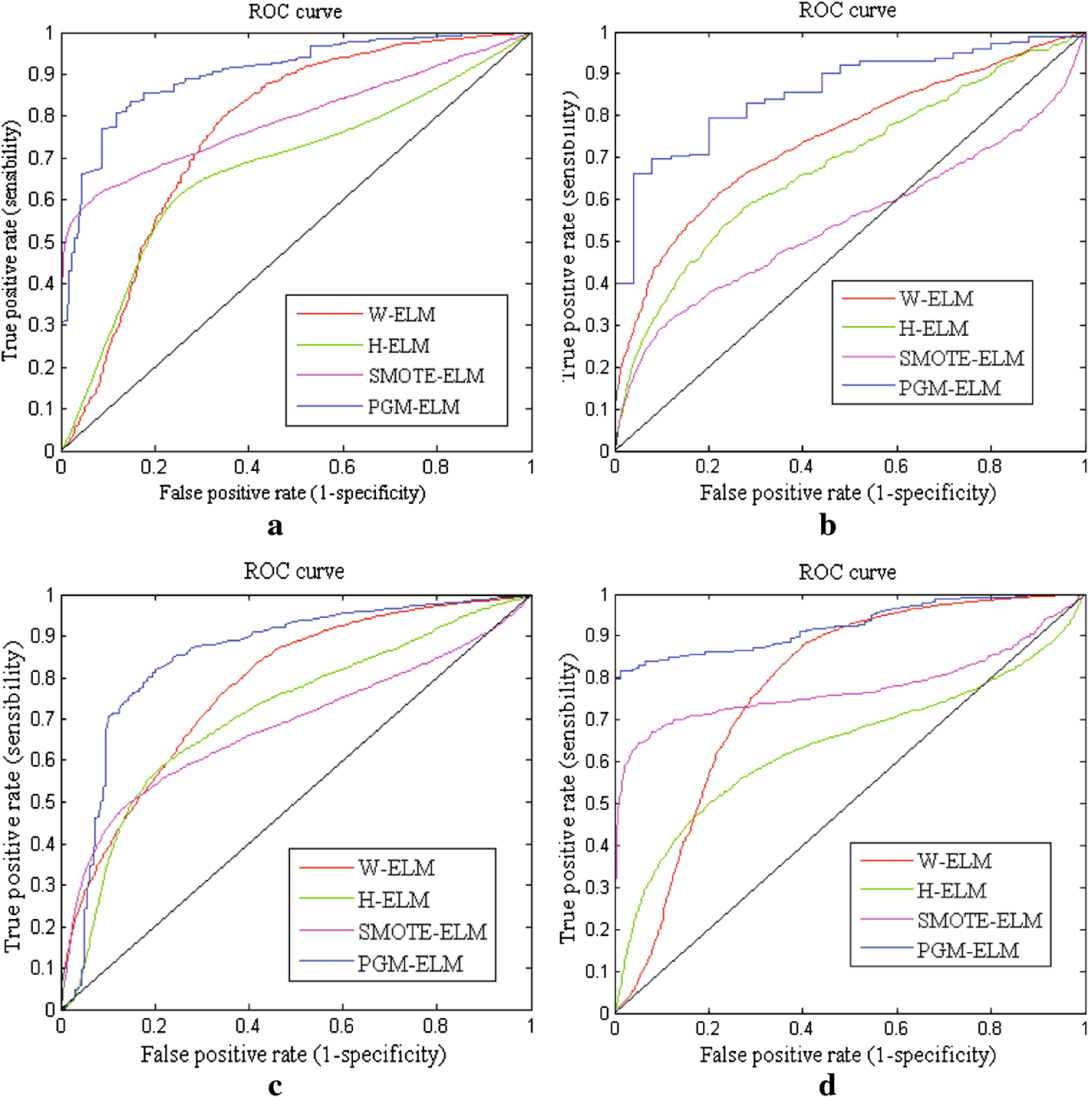
Comparison ROC curves of PGM-ELM, H-ELM, SMOTE-ELM, and W-ELM. **a** Breast cancer dataset. **b** Liver patient dataset. **c** Diabetic retinopathy dataset. **d** Pima diabetes dataset

Meanwhile, we assess the classification performance of four models in terms of ROC curve. Figure 7 shows comparison results of the averaged ROC curve on four datasets. From almost most of results of Fig. 7a–d can be seen that, by comparing with other three algorithms, the PGM-ELM method has much higher ROC curve on each dataset. However, H-ELM has a relatively poor performance, especially on small training set, which is showed in Fig. 7a, d. It can explain that H-ELM is sometimes difficult to control the optimal hyper-parameters by manually tuning parameter. In Fig. 7b, the ROC curve of SMOTE-ELM is higher at first and tends to the obvious decline at last. Generally, SMOTE method uses local information to generate synthetic samples. When the training set is smaller and severe imbalanced, it usually ignores the overall class distribution, leading to some information loss. By contrast, although W-ELM reveals a merely superior recognition ability to these two algorithms on breast, liver, and diabetes datasets. But if data dimensionality is greater, W-ELM poorly performs the classification due to some redundant features. The PGM-ELM can present better performance thanks to the realistic-looking samples generation and the information loss reduction by dynamic GAN. More importantly, biomedical hidden features are learned by using layer wise unsupervised learning.

**Fig. 8:**
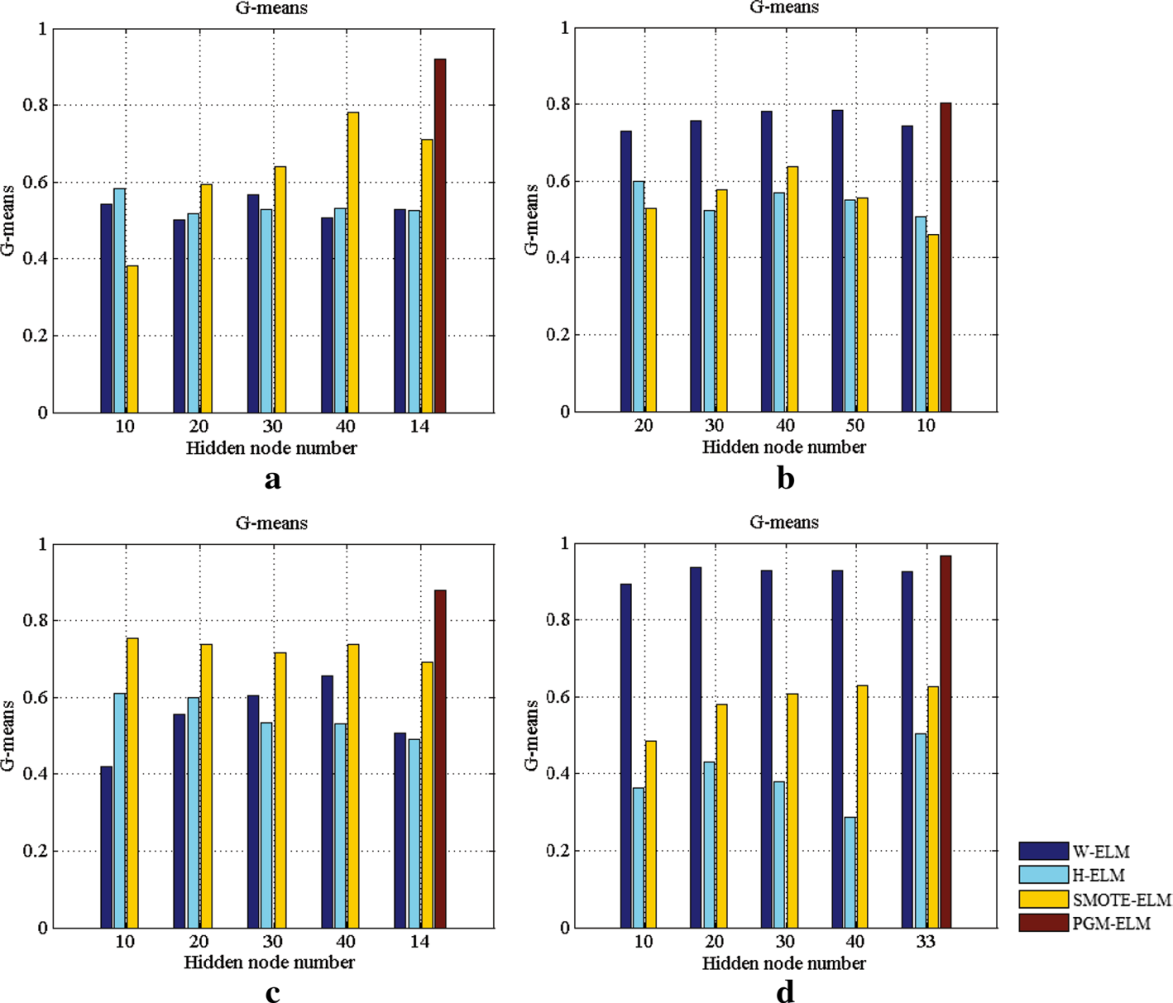
Comparison G-means of the PGM-ELM, H-ELM, SMOTE-ELM, and W-ELM with different numbers of hidden nodes. **a** Breast cancer dataset. **b** Liver patient dataset. **c** Diabetic retinopathy dataset. **d** Pima diabetes dataset

**Fig. 9:**
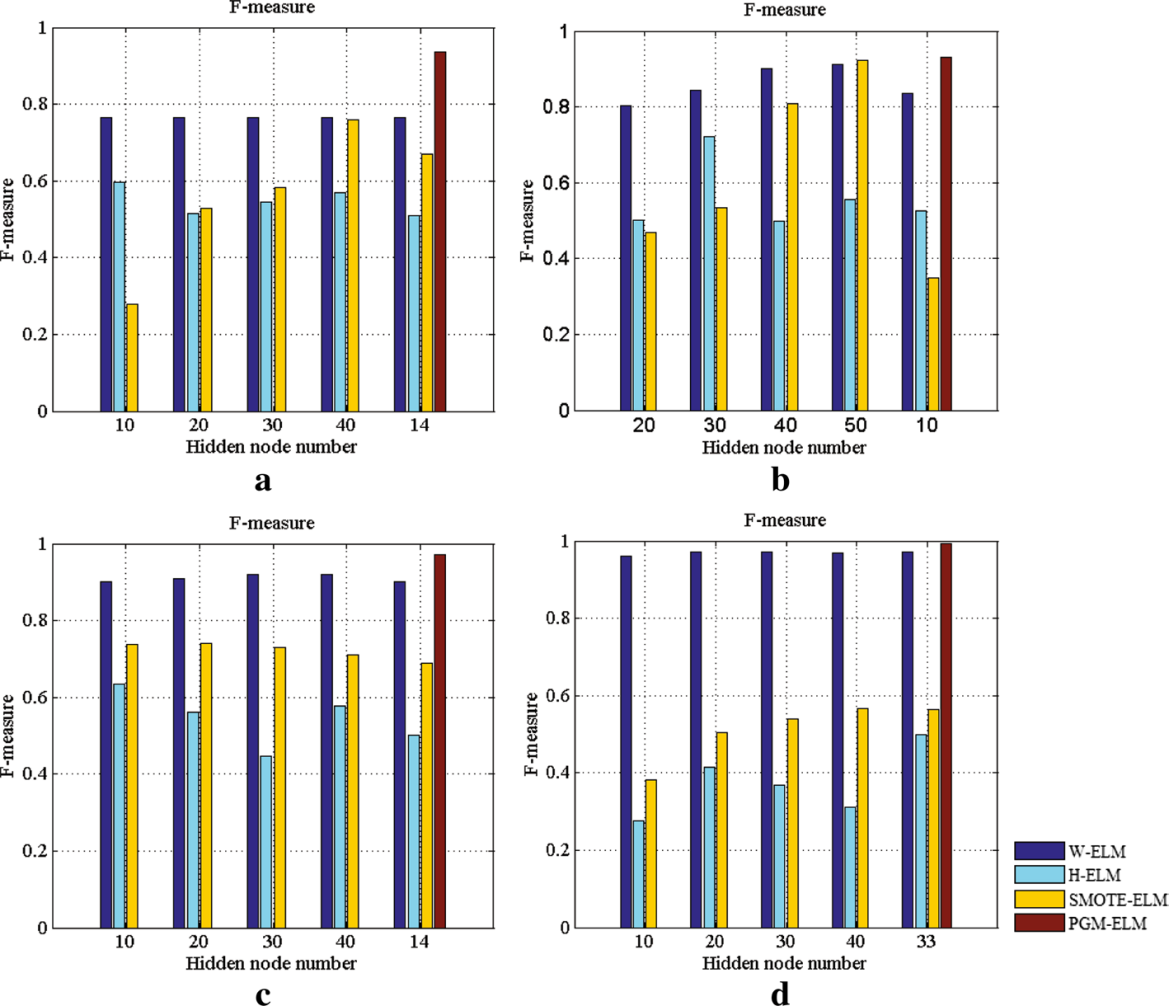
Comparison F-measures of the PGM-ELM, H-ELM, SMOTE-ELM, and W-ELM with different numbers of hidden nodes. **a** Breast cancer dataset. **b** Liver patient dataset. **c** Diabetic retinopathy dataset. **d** Pima diabetes dataset

Now onto a discussion about the number of hidden nodes in ELMs model. Limited availability of the training samples necessitates careful selection of the parameters of the hidden layer, thereby achieving well-generalizing model. To this end, we give the analytic expression for numbers of layer and hidden node in PGM-ELM. The accumulated G-means and F-measures of four models as changing the number of hidden nodes are illustrated in Figs. 8 and 9.

As can be seen from Figs. 8a and  9a, taking breast cancer dataset as an example, the PGM-ELM gets the highest G-mean and F-measure when the number of hidden nodes is 14. It suggests that our method obtains better classification accuracy and robustness. Besides, we can easily observe that, compared with H-ELM, PGM-ELM shows superior performance in case of same number of hidden nodes on most of datasets. This indicates that PGM-ELM is not sensitive to the hyper-parameter of hidden layer by considering the changes of imbalance ratio and sample distribution. This is explained by the fact that the analytical solution for parameters of the hidden layer makes classification results more accurate. For W-ELM and SMOTE-ELM, G-mean and F-measure only slightly change with different hidden nodes. This is perhaps because that simpler single layer network is also less sensitive to the number of hidden nodes. As a consequence, these results demonstrate the adaptability of the proposed PGM-ELM in dealing with small sample and imbalanced data.

## Discussions

In this study, we have developed a self-adaptive multilayer ELM model combining with dynamic GAN to classify the limited and imbalanced dataset for the biomedical engineering application. Representative W-ELM, SMOTE-ELM, and H-ELM models are also implemented to solve the biomedical data classification in our work. In this section, we discuss the classification performance, the statistical significance, and the computational time of these four models. At last, the advantages and limitations of the PGM-ELM method are summarized.

### Evaluation of the classification performance

To further objectively verify the superiority of the proposed method, extensive evaluations are conducted on four datasets. We compute G-mean, AUC, and F-measure metrics of four methods. Table 4 tabulates the quantitative comparison results of different methods on four biomedical datasets in terms of G-mean, F-measure, and AUC.

**Table 4:** Performance comparison results of testing on different datasets

Biomedical datasets	Methods	G-mean	AUC	F-measure
Breast cancer	W-ELM	0.5679	0.8093	0.7658
	SMOTE-ELM	0.7816	0.7981	0.7584
	H-ELM	0.5835	0.6584	0.5967
	PGM-ELM	0.9212	0.9013	0.9354
Liver patient	W-ELM	0.7827	0.7439	0.9127
	SMOTE-ELM	0.6379	0.5198	0.9218
	H-ELM	0.5980	0.6919	0.7226
	PGM-ELM	0.8016	0.8581	0.9304
Diabetic retinopathy	W-ELM	0.6555	0.7849	0.9207
	SMOTE-ELM	0.7554	0.7220	0.7404
	H-ELM	0.6112	0.7493	0.6346
	PGM-ELM	0.8778	0.8619	0.9715
Pima diabetes	W-ELM	0.9360	0.9151	0.9724
	SMOTE-ELM	0.6277	0.8792	0.5655
	H-ELM	0.5041	0.8580	0.5000
	PGM-ELM	0.9657	0.9324	0.9922

From the AUC values in Table 4, we can clearly observe through the comparison and analysis, the proposed PGM-ELM has a much larger value than SMOTE-ELM and H-ELM, while a little higher than W-ELM for most of the test sets. The reason calls for PGM-ELM, the input of the model is changed from the original imbalanced data to a more balanced one by dynamic GAN. From the values of G-mean and F-measure, we also can find that our approach has a significant improvement against the others on four datasets. Especially, for Pima diabetes dataset, the value of F-measure nearly tends to 1. The hyper-parameter analytic expression of hidden layer helps to achieve a better performance by providing more robust features extract from the balanced data. Meanwhile, an important observation is that fewer parameters need to be chosen adaptively in the training process. The whole performance of the algorithm is not only high but also stable.

### The statistical significance testing

In the statistical hypothesis testing, the Friedman test and post-hoc Nemenyi test [38] are used to further analyze whether our method is statistically significant than other compared methods. Combining these two hypothesis testing methods are to compare the performances of various classification methods on multiple datasets. After Friedman hypothesis testing, the null hypothesis (i.e. the performances of all four methods are equivalent) is rejected at $\alpha =0.05$ since the *p*-values for G-mean, AUC, and F-measure are 0.0256, 0.0129, and 0.0112. This result indicates that our method has a significant difference than the others.

Then, the post-hoc Nemenyi test is adopted to observe the differences among the four models. A critical difference (CD) of 2.345 is computed at $p = 0.05$. For G-mean metric, the average ranks of PGM-ELM, W-ELM, SMOTE-ELM, and H-ELM are 1, 2.75, 2.5, and 3.75, respectively. From these rank differences among PGM-ELM, W-ELM and SMOTE-ELM, they are lower than the CD value. So PGM-ELM has no statistically significant difference in terms of G-mean, despite our method wining on most of the datasets. While PGM-ELM is statistically different from H-ELM. This explains why our method is suitable for the imbalanced data classification problem.

### Comparison of the computational time

**Fig. 10:**
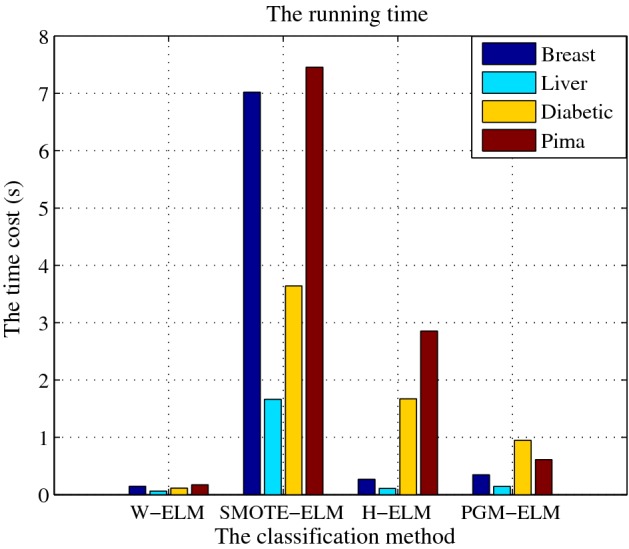
Comparison result of the running time

The classification efficiency of the W-ELM, SMOTE-ELM, H-ELM, and PGM-ELM algorithms are compared, which is presented in Fig. 10. By analyzing the computational times, we can find that the training time of PGM-ELM is slightly higher than that of W-ELM. And it is obviously lower than those of H-ELM and SMOTE-ELM. The reason for this is that a lot of time is costed for the sample generation process using GAN. W-ELM has a computational advantage owing to its fast weighting process. Nevertheless, if the imbalanced ratio is extremely low, the W-ELM usually leads to an excessive learning. It is difficult to control the optimal parameter. Anyway, the computational time of PGM-ELM method on each dataset is below 2s. In a word, the proposed method can quickly and accurately alleviate the class-imbalanced problem. These findings demonstrate that the algorithm presented here has a potential significance for the clinical practice.

Based on the above analysis, we can summarize the advantages and limitations of the proposed method. Our method attempts to tackle the classification of limited and imbalanced biomedical dataset. In the proposed method, dynamic GAN takes the data distribution into account for producing authentic minority class samples. Furthermore, the parameters of hidden layer are adaptively chosen according to the change of the imbalanced ratio. It avoids the drawback of manual parameter adjustment. Under imbalanced scenarios, different types of biomedical data (e.g. protein dataset, gene expression data, and medical images) have similar properties, such as high-dimensional and small samples. For example, image data can be converted to numerical attributes by using some segmentation methods [39, 40]. In this way, the proposed method can effectively address the class-imbalanced classification problem with respect to different biomedical datasets. Despite this goodness, the proposed method has also two potential weakness. One limitation is that the time cost of our method is slightly higher than W-ELM, mainly due to extra cost of the samples generation process. The other is, if a large of missing values occur in biomedical dataset, GAN model will generate some ineffective samples. The proposed model also will suffer from worse classification performance. In future works, these two limitations will be addressed.

## Conclusions

In this paper, a self-adaptive multilayer ELM with dynamic GAN has been proposed for the imbalanced biomedical classification. Different from traditional deep network, self-adaptive multilayer ELM gives the analytic expression for numbers of layer and hidden node according to the changes of the imbalanced ratio and sample distribution. This is helpful for avoiding the hyper-parameter sensitivity. Furthermore, principal components of the original features are extracted by PCA, thus removing irrelevant features and obtaining more effective feature set. Then, dynamic GAN generates the real-looking samples to balance the class distribution. It fully considers the sample distribution and reduces overfitting. The proposed method has been evaluated on four real-world biomedical datasets. Qualitative and quantitative results show that the proposed method is quite promising than other representative methods in terms of ROC curve, AUC, G-mean, and F-measure metrics. The generality and capability of the proposed model are further confirmed under the condition of small sample and high-dimensional feature. We will make efforts to provide multi-class classification model for multiclass imbalanced classification problem in our future works.

## Abbreviations

CAD: computer-aided diagnosis
ELM: extreme learning machine
W-ELM: weighted extreme learning machine
H-ELM: hierarchical extreme learning machine
EUS: evolutionary undersampling
SMOTE: synthetic minority oversampling technique
GAN: generative adversarial nets
PCA: principal component analysis
ROC: receiver operator characteristic
AUC: area under the ROC curve
